# Infants' Visual Preference for Upright Faces During the COVID‐19 Pandemic in Japan

**DOI:** 10.1111/infa.70052

**Published:** 2025-11-19

**Authors:** Nobu Shirai, Mizuki Kawai, Yumiko Otsuka, Megumi Kobayashi, Tsunehiko Tanaka

**Affiliations:** ^1^ Department of Psychology Rikkyo University Tokyo Japan; ^2^ Clinical Psychology Course Graduate School of Modern Society and Culture Niigata University Niigata Japan; ^3^ School of Psychology Chukyo University Aichi Japan; ^4^ Department of Psychology Faculty of Humanities Niigata University Niigata Japan; ^5^ Department of Educational Psychology Faculty of Education Niigata University Niigata Japan

**Keywords:** COVID‐19 pandemic, face perception, infant, masked face

## Abstract

The coronavirus disease 2019 (COVID‐19) pandemic required face masks to be worn in public spaces, changing daily face‐to‐face communication. This study investigated whether infants perceived masked faces as faces during the COVID‐19 pandemic, a time when wearing masks in public was nearly universal in Japan. Between July 2021 and November 2022, we conducted an experiment using a novel remote method to examine infants' preferences for upright versus inverted faces under both masked and unmasked conditions. Overall, infants aged 4–5 months and 7–8 months (*n* = 32 in each age group) preferred upright to inverted faces in both conditions. These results suggest that Japanese infants born during the COVID‐19 pandemic could recognize masked faces as faces. Furthermore, across both age groups, the preference was more pronounced in the masked‐face condition than in the unmasked‐face condition. These results possibly reflect the fact that infants had relatively frequent exposure to masked faces during the pandemic.

## 1 Introduction

Significant and potentially serious changes in facial perception have occurred worldwide in recent years. In March 2020, the World Health Organization declared that the outbreak of the novel coronavirus disease 2019 (COVID‐19) had become a global pandemic (World Health Organization 2020). As a result, changes in daily behaviors, such as refraining from unnecessary outings, engaging in frequent hand washing and hand sanitization, and restrictions on eating out and large gatherings, occurred worldwide. Moreover, people were advised to wear masks in public settings to prevent the disease from spreading. In particular, wearing masks in public places and social situations was strongly recommended in Japan (Prime Minister’s Office of Japan 2020), leading to many Japanese people covering the lower half of their faces with masks during the COVID‐19 pandemic.

Concerns have emerged regarding the impact of mask wearing on the development of face perception (for a review, see Carnevali et al. 2022). Several empirical studies have shown lower performance in recognition tasks for faces wearing masks than for faces not wearing masks (e.g., Freud et al. 2020). This phenomenon has been observed in both adults and school‐aged children (Carbon and Serrano 2021; Stajduhar et al. 2022).

Prior to the pandemic, studies have shown that infants, even newborns, preferred upright faces and face‐like patterns over inverted configurations (Morton and Johnson 1991; Valenza et al. 1996; Macchi Cassia et al. 2004; Macchi Cassia et al. 2006; Otsuka et al. 2012). This preference has been interpreted as evidence of face detection and perception by infants (e.g., Doi et al. 2009; Kobayashi et al. 2012; Yamanaka et al. 2025). In newborns, the upright face preference can be triggered by simple visual features, such as top‐heavy patterns, and not necessarily by canonical facial configurations (Simion et al. 2002; Macchi Cassia et al. 2004). These results suggest that newborns have only a crude representation of the facial structure. Over the first few months, upright preference becomes more specific to canonical face configurations (e.g., Macchi Cassia et al. 2006; Chien 2011), indicating the development of more refined facial representations. Around the same time, infants begin to show preferences for faces of the same gender as their primary caregiver and of their own race (e.g., Quinn et al. 2002; Kelly et al. 2005, 2007), suggesting that daily visual experience shapes infants' face representation.

Research on face recognition emphasizes the role of global configural processing in the development of adult‐like face expertise. These include sensitivity to canonical spatial relationships between facial features, holistic integration of features, and sensitivity to the spacing between them (Maurer et al. 2002). Disruptions to the canonical face configuration, such as face inversion, impair both holistic processing and sensitivity to spacing (Maurer et al. 2002). Although some evidence suggests that configural processing exists from birth (e.g., Leo and Simion 2009), most studies indicate that it develops gradually throughout infancy and childhood (Thompson et al. 2001; Bhatt et al. 2005; Hayden et al. 2007; de Heering and Schiltz 2013).

As face masks cover the lower half of the face, they disrupt the canonical configuration of facial features. Thus, it is not surprising that recognition of masked faces was impaired. Given the ubiquity of wearing masks during the pandemic, infants' exposure to full facial configurations is likely to be reduced, especially outside the home.

Recent studies have examined the effects of masks and the pandemic on infants' face processing. Galusca et al. (2023) reported age‐related changes in upright face preference for masked faces: 3–6‐month‐olds showed no clear preference, whereas 9–12‐month‐olds preferred inverted masked faces, suggesting atypical responses. DeBolt and Oakes (2022) found that 6‐ to 9‐month‐old infants showed novelty preferences only when tested with unmasked faces, regardless of whether the familiarized face was masked. Kim et al. (2024) compared face recognition in infants aged 8 and 13 months. When familiarized with a frontal face and tested with quarter‐view faces, younger infants showed novelty preference, but older infants did not, in contrast to pre‐pandemic findings reporting successful face recognition around this age (e.g., Kelly et al. 2007, 2009; Chien et al. 2016). In a second experiment, only older infants showed novelty preference for masked faces, suggesting that increased exposure to masked faces enhanced recognition.

A neuroimaging study examined the effects of the pandemic on infant brain responses to human faces. Yates et al. (2023) used fMRI to compare infant brain responses to faces before and after the pandemic. Pre‐pandemic infants showed typical repetition suppression in the fusiform face area (FFA), whereas post‐pandemic infants showed increased activation in response to repeated faces. These results suggest that altered visual experiences, including mask‐wearing, affect the development of facial processing.

The current study was conducted in Japan during the COVID‐19 pandemic, between July 2021 and November 2022, when mask‐wearing in public was nearly universal. Our goal was to examine whether infants perceive masked faces as faces. Previous studies have suggested that face recognition abilities become increasingly specialized during infancy, based on daily experiences with faces. As a result, toward the end of the first year of life, face recognition performance is optimized for faces within one's own cultural environment (i.e., faces of one's own species and race) (Pascalis et al. 2002; Kelly et al. 2007; Scott et al. 2006; Kelly et al. 2009). If infants could not recognize masked faces as faces, it would suggest that they had severely limited opportunities for facial learning outside their households during the pandemic. Conversely, if they could, they may have made the best use of the limited visual input from the visible parts of the masked faces to develop their face‐processing abilities, even during the pandemic. Therefore, it is important to investigate whether infants perceive masked faces as faces during a pandemic.

To test whether infants perceived masked faces as faces, we measured their preference for upright faces over inverted faces in the unmasked‐ and masked‐face conditions. To the best of our knowledge, this is the first study to directly compare upright face preferences between masked and unmasked faces in infancy. In the unmasked condition, the infants viewed pairs of upright and inverted unmasked faces, whereas in the masked condition, they viewed pairs of upright and inverted masked faces. If they recognized masked faces as faces, they would show an upright face preference under both conditions. Otherwise, the preference would appear only in the unmasked condition. This design allowed us to infer whether the masked faces were perceived categorically as faces by infants.

We also examined age‐related changes in upright face preference by comparing two age groups: 4–5 months and 7–8 months. The younger group was selected based on the finding that the upright preference becomes more specific to canonical facial structures around this age (Chien 2011). The older group was chosen because an upright preference has been reported at 8 months (Dobkins and Harms 2014). We hypothesized that older infants would show a stronger preference for upright faces in the masked condition, assuming that they were more exposed to masked faces. During data collection, mask‐wearing was widespread in public spaces (Prime Minister’s Office of Japan 2020) but was rarely practiced at home. Since younger infants (e.g., 3‐month‐olds) primarily see faces at home (Sugden and Moulson 2019), their face exposure may not have changed compared to the pre‐pandemic period as drastically as older infants. Older infants were likely to have more opportunities to go outside and see masked faces during the pandemic, although this may vary by family. If experience is critical for recognizing masked faces as faces, older infants should show a stronger upright face preference than younger infants. Another possible reason for the developmental changes is the improvement in visual completion ability with age. Studies have suggested that the ability to represent occluded objects improves in the latter half of infancy (Craton 1996; Otsuka et al. 2006). This may allow older infants to perceive the continuation of the facial structure behind the occluding mask when seeing a masked face, thereby helping them recognize the masked face as a coherent face.

In summary, we examined upright face preference in infants aged 4–5 and 7–8 months using masked and unmasked faces. Owing to pandemic safety concerns, we used an on‐demand remote testing system (Shirai et al. 2022), and all data were collected by parents at home using a tablet device.

## 2 Methods

### 2.1 Ethics Statement

The study protocol was approved by the Ethics Committee of Niigata University (2020‐0166, 2022‐0003). The study was conducted in accordance with the Declaration of Helsinki. Written informed consent was obtained from the parents of infants who participated in this study.

### 2.2 Participants

The final sample consisted of 64 infants: 32 aged 4–5 months (15 girls; mean age = 143.3 ± 21.5 days) and 32 aged 7–8 months (15 girls; mean age = 228.3 ± 15.7 days). All infants were born at term with a birth weight of ≥ 2500 g. Since no previous study has measured infants' visual preference for masked faces, it was difficult to estimate the appropriate sample size. Therefore, we determined the sample size using the following heuristic approach (Lakens 2022). A sample size of ≥ 20 per experimental group has been recommended in psychological research to reduce the risk of type I error (Simmons et al. 2011; see also Oakes 2017, for an infant study). Because we needed to counterbalance the 16 combinations of stimuli (two experimental sessions × two initial face positions × four face models; see the Procedure section for details), the sample size was required to be a multiple of 16. Thus, 32 participants were recruited from each group.

An additional 32 infants (22 aged 4–5 months and 10 aged 7–8 months) were excluded from the final sample. Among these infants, nine were excluded because their viewing distance during the experiment was too short or long, or their gaze was not appropriately recorded in video images; seven because of blurring or occlusion in video images (caused by parents' errors in attaching a conversion lens to a tablet); six because they failed to complete all experimental sessions; three because the experimental sessions were conducted over multiple days, not in accordance with the instructions; three because of an equipment failure (non‐presentation of “beep” sound stimulus or software application shutdown before completion of the session); one because of parental interference (forcefully orienting the infant's head toward the tablet computer screen); one because the parent reported that “almost all people [their infant had encountered] were not wearing masks”; and two because of outliers discovered during data coding. One of these latter two participants had an extremely short total time looking at visual stimuli across all four 20‐s trials compared with that of the overall sample (7.96 vs. 50.36 ± 11.33 s; difference of > 3 standard deviations), and the other was excluded because of an extreme (100%) bias toward the right side of the screen across all experimental trials.

All participants were recruited via flyers distributed by the public health authority of Niigata City and through advertisements placed in free newspapers and posted on the websites of the authors' laboratories (MKo and NS). Thus, the participants in the present study were recruited primarily from Niigata City and its vicinity, as well as from various regions across Japan. Moreover, all participants were Japanese and came from families with a middle‐class socioeconomic background in Japan.

### 2.3 Apparatus

**FIGURE 1:**
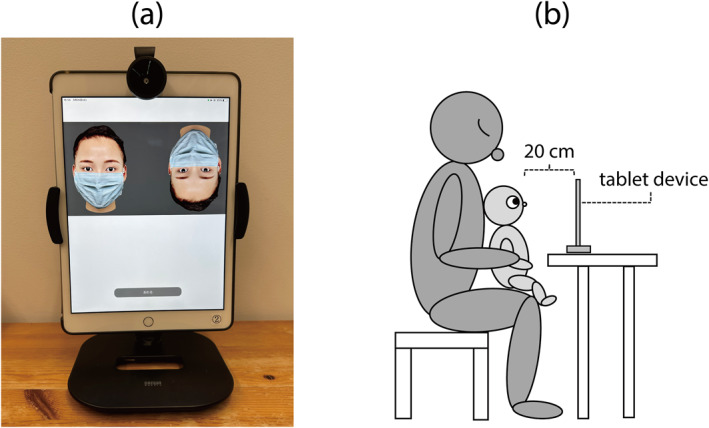
(a) Photograph of the equipment used in this study. (b) Schematic showing the positional relationship among an infant, her/his parent, and the experimental equipment.

The apparatus used in this study is shown in Figure 1. Tablet devices (seventh‐ or eighth‐generation 32‐GB iPads; Apple Inc., Cupertino, CA, USA) were used to display the visual stimuli and record participants' responses. Customized applications were created using Swift in Xcode Integrated Development Environment (ver. 12.4 and ver. 13.0 for the seventh‐ and eighth‐generation iPads, respectively), controlled the presentation of stimuli, and were used to record the infants' looking responses (via the front camera of the tablet). The tablet was placed in front of the participant on a stand (100‐LATAB013 W; Sanwa Supply, Okayama, Japan) in a portrait orientation. A wide‐angle conversion lens (P‐SL04BK; Elecom Co., Osaka, Japan) was attached to the front camera of the tablet to ensure that the entire face of each infant could be captured, even in the event of movement and postural changes.

### 2.4 Stimulus

Each stimulus was converted into MP4 format for presentation on the tablet device screen. The original resolution of the MP4 files was 1920 × 1080 pixels (Px), rescaled to the native resolution (1620 × 911 Px) for presentation on the tablet screen. All stimuli appeared on the upper half of the screen (Figure 1) to ensure that the infants' eyes remained fixed immediately below the tablet's camera.

**FIGURE 2:**
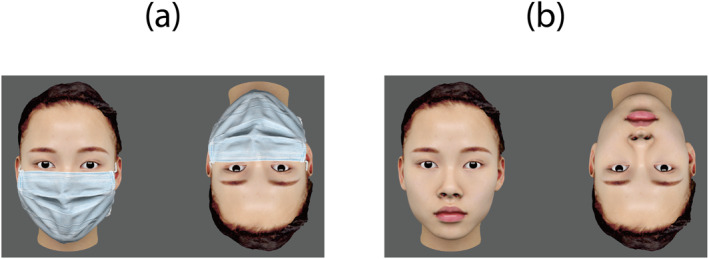
Example visual stimuli. (a) Masked and (b) unmasked female faces.

Each visual stimulus consisted of an upright face and an inverted face appearing side‐by‐side on a uniform gray background. The two faces were identical, except for their orientation (upright vs. inverted; Figure 2). The faces were 15.3° wide and 23.2° high, and the distance between the two faces in each pair was 11.2°.

We used four East Asian faces (two men and two women with neutral facial expressions) from commercially available software (3D Scan Store; https://www.3dscanstore.com/) to generate face images without masks. Using a 3D computer graphics software (Blender, ver. 2.79; https://www.blender.org/), we inserted 3D eyeball models into each head (for details, see Otsuka et al. 2015) and fitted the 3D hair models. The gaze direction of the eyeballs was set in the frontal direction. A 3D model of a widely used mask (Creazilla; https://creazilla.com/nodes/1795190) was used to create the masked‐face images. We fit the 3D model of the mask to each 3D face model using Blender's cloth simulation function. Subsequently, with the mask fitted, each face was rendered in Blender to produce the final masked‐face images. The only difference between masked and unmasked facial images was the presence or absence of a mask.

### 2.5 Procedure

Parents who applied for the study were sent instruction leaflets, a consent form, and the study equipment (tablet computer, wide‐angle conversion lens, and tablet stand) through a parcel delivery service. The parents were asked to set up the equipment on a stable desk in a quiet location. No specific instructions were provided regarding lighting. During the experiment, each parent sat in front of the tablet with their infant on their lap. Before the experiments began, the parents adjusted the distance between the tablet and infant's face to 20 cm (see Figure 1). Because the application used to run the experiment had no specific function for automatically measuring the viewing distance, the parents were instructed to measure the distance between the tablet and the infant's face and to keep the viewing distance as consistent as possible during the experiment. When the application was launched, a calibration panel with a small window showing the video input in real‐time appeared on the screen. The parent adjusted the position of the infant relative to the tablet, ensuring that the infant's face was in the center of the window. When the parent touched the “Start” button in the lower part of the calibration panel, the experimental session began. The parents were asked not to look at the tablet screen during the experiment and instructed to either look in a different direction or close their eyes. Each experimental session comprised two trials.

An “attention grabber” (a colorful cartoon character accompanied by a beep sound) was displayed in the center of the screen for 5 s, and the first visual stimulus (an upright and inverted face pair) was then presented for 10 s. The attention grabber reappeared for 5 s immediately after presentation of the first stimulus, followed by the second stimulus (10 s). The positions of the upright and inverted faces were reversed in the first and second trials. The timing of the transition between the attention grabber and visual stimuli and the termination of the experimental session were automatically controlled by the application. Thus, no manipulation of the tablet device was required after the parent pressed the “Start” button. Each infant completed one experimental session with masked face stimuli and the other with unmasked face stimuli. Each infant completed four trials. The order of the two experimental sessions was counterbalanced across the participants. The positions of the upright and inverted faces in each trial were determined based on the “ABBA” rule: if an upright (inverted) face appeared on the right (left) side of the screen in the first trial, then an inverted (upright) face appeared on the right (left) in the second and third trials, and the positions of the faces were swapped again in the final trial. The positions of the upright and inverted faces in the experimental trials were counterbalanced across the infants. Moreover, each infant was presented with only the faces of one of the four models, and the model was counterbalanced across infants. Thus, 16 combinations were counterbalanced across infants (2 experimental sessions × 2 initial face positions × 4 face models).

We also asked the parents to estimate their infants' degree of experience with masked faces via the following question: “What proportion of the individuals (including family members) your infant has encountered were wearing masks? Please select the most suitable option from the following: (1) None of them were wearing masks; (2) The majority of them were not wearing masks; (3) About half of them were wearing masks; (4) The majority of them were wearing masks; (5) Almost all of them were wearing masks.” Owing to a lack of experience with masked faces, we excluded one participant who selected option (1) from the final sample analysis (see the Participants section).

After the two experimental sessions were completed, the parents sent a signed consent form and the study equipment back to the laboratory using a parcel delivery service. The shipping costs were covered by the authors.

### 2.6 Data Coding

One of the authors (MKa; main coder) coded videos of the infants' looking behaviors using a custom event recorder developed in Processing software (ver. 3.5.4; Processing Foundation, New York, NY, USA) and a personal computer. The coder was blinded to the stimulus type and position during the coding of each video. The coder pressed the assigned keys on a computer keyboard according to whether the infant's gaze was oriented toward the right or left side of the tablet screen. The key was depressed as long as the infant looked at the corresponding part of the screen; this was recorded as the looking time. No keypress was made if the infant did not look at the screen. Another author (NS; sub‐coder) coded half of all the videos in the same manner to check the validity of the results of the main coder. There was a strong correlation (*r* = 0.960) between the coding results (512 results: four trials × two screen areas [right vs. left] × 32 randomly chosen participants) of the main coder and sub‐coder, and the results of the main coder were used for statistical analysis.

## 3 Results

### 3.1 Main Analyses

**FIGURE 3:**
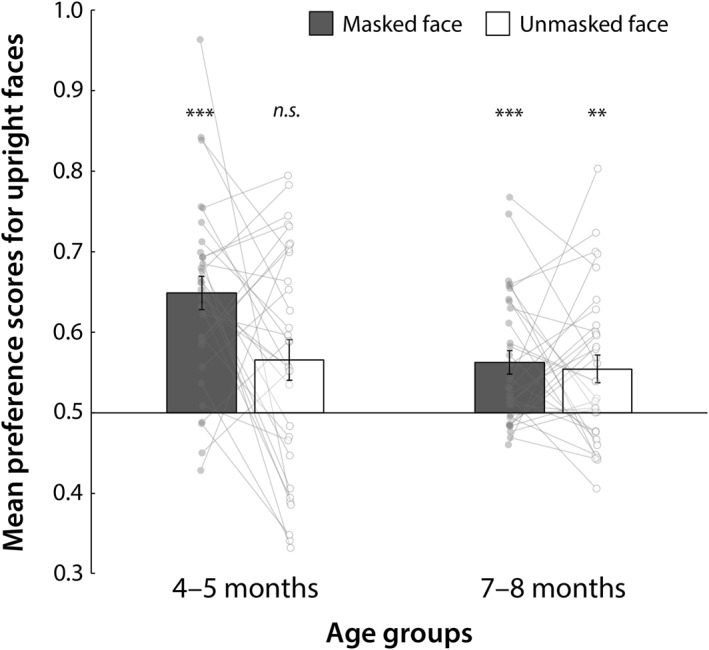
Mean infant preference scores for upright face stimuli. The left and right sides of the graph show the results for the groups aged 4–5 months and 7–8 months, respectively (both *n* = 32). Dark and white bars represent preference scores in the masked‐ and unmasked‐face conditions, respectively. Error bars indicate standard errors of the mean. Spaghetti plots show the results of individual participants.

We calculated the upright face preference score for each infant and condition by dividing the looking time for the upright faces during the two test trials by the total looking time for the two trials. The mean scores for each age group and face type (masked vs. unmasked) are shown in Figure 3. The results are summarized in Table 1.

**TABLE 1:** Preference scores of each infant for the masked and unmasked face conditions and answers to the questionnaire.

Preference score for masked faces	Preference score for unmasked face	Answer to questionnaire
4–5 months		
0.7154665	0.60195412	5
0.68518663	0.66209435	5
0.65643288	0.44209803	4
0.69096152	0.74614684	5
0.84203384	0.69530274	5
0.588688	0.705694	4
0.54010613	0.33606847	4
0.42370604	0.71470034	4
0.59698835	0.46617067	4
0.66600707	0.38440729	5
0.84583797	0.62206242	5
0.75281309	0.79593426	5
0.63355182	0.55401963	4
0.66492195	0.53055356	5
0.64617907	0.55243295	5
0.51013354	0.46383274	5
0.67111539	0.4007094	5
0.62307489	0.59479595	5
0.73960111	0.58415638	5
0.67243939	0.78610682	5
0.68313302	0.47882695	5
0.58082396	0.71216434	4
0.69119887	0.73529412	3
0.48503377	0.59264574	5
0.55388397	0.6559484	5
0.44688243	0.55439953	4
0.58632343	0.73476325	5
0.70330727	0.32744028	4
0.63504643	0.38993922	4
0.75908336	0.54728647	5
0.96763473	0.38068141	4
0.4893948	0.34379025	4
7–8 months		
0.58764957	0.53670257	3
0.49584181	0.44545113	4
0.66082637	0.49636666	5
0.47535604	0.50614908	5
0.52122271	0.58098443	3
0.65785229	0.70123061	5
0.63058677	0.59946887	4
0.55734119	0.63042019	5
0.57375285	0.46300152	4
0.50862069	0.61123897	5
0.66689933	0.43676752	4
0.49638005	0.50652061	4
0.52653288	0.80734717	3
0.5185534	0.47465216	4
0.65185041	0.72581969	5
0.77012467	0.67669513	5
0.50862305	0.60099649	5
0.64116447	0.51419403	5
0.48177771	0.57124103	4
0.61417722	0.44000689	5
0.48200888	0.51561205	5
0.45604951	0.70182761	5
0.51003093	0.49947209	5
0.64446809	0.45535444	5
0.52783779	0.64328317	5
0.52254346	0.40193664	4
0.46974224	0.44182712	5
0.75062247	0.55647326	4
0.48011236	0.58456515	5
0.48206158	0.59943522	5
0.58348422	0.53654878	5
0.54377832	0.47369894	5

Two‐tailed one‐sample *t*‐tests with Bonferroni correction (*α* = 0.05/4) revealed that infants aged 4–5 months had a significant preference for upright faces in the masked‐face condition (t [31] = 7.18, *p* < 0.001, *g* = 1.77), while those aged 7–8 months had a significant preference for upright faces in both the masked‐ and unmasked‐face conditions (t [31] = 4.22, *p* < 0.001, *g* = 1.04; and t [31] = 3.13, *p* = 0.004, *g* = 0.77, respectively). The preference for upright faces among 4–5‐month‐olds in the unmasked‐face condition did not reach statistical significance following Bonferroni correction (t [31] = 2.61, *p* = 0.056, *g* = 0.64). However, it is noteworthy that the magnitude of this effect was medium to large and comparable to the preference observed in the 7–8‐month‐old group under the same conditions (*g* = 0.77).

Two‐way mixed analysis of variance (ANOVA; 2 age groups [4–5 vs. 7–8 months] × 2 face types [masked vs. unmasked] revealed significant main effects of age (F [1, 62] = 5.75, *p* = 0.020, ηG2 = 0.16) and face type (F [1, 62] = 5.49, *p* = 0.022, ηG2 = 0.04) on upright face preference scores, although there was no significant age × face type interaction effect (F [1, 62] = 3.69, *p* = 0.059, ηG2 = 0.03). These results indicate that infants in both age groups generally showed a stronger preference for the upright face in the masked condition than in the unmasked condition, and younger infants showed a greater upright face preference than older infants.

### 3.2 Additional Analyses

To analyze the relationship between the reported daily experience of masked faces and the results of the preferential looking experiment, Kendall's rank correlation was calculated between the answers to the questionnaire items pertaining to infants' degree of experience with masked faces and upright face preference in each of the two face conditions (masked and unmasked). The correlation was not significant for either 4–5‐month‐olds (masked face: *τ* = 0.248, *p* = 0.091; unmasked face: *τ* = 0.204, *p* = 0.166) or 7–8‐month‐olds (masked face: *τ* = −0.058, *p* = 0.689; unmasked face: *τ* = 0.124, *p* = 0.391). However, the majority of parents chose answer option 4 or 5, and all parents in the final sample chose options ≥ 3 (Table 1), indicating little variation in the answers. As the primary purpose of the questionnaire was to screen out participants who had no experience of seeing masked faces, the results may not sensitively reflect the participants' actual experience with masked faces. Therefore, these non‐significant correlations were unsurprising.

We also compared upright face preferences between the younger and older age groups using a statistical test different from the main analyses. This additional analysis aimed to provide further insight into the potentially inconsistent results obtained from the two main analyses. In one of the main analyses (one‐sample *t*‐tests), the younger infant group showed a significant upright face preference only in the masked‐face condition, whereas older infants showed a significant upright face preference in both the masked and unmasked face conditions. In the other main analysis (two‐way ANOVA), however, we found significant main effects for both age and face condition, without a significant interaction between them. Therefore, unlike the *t*‐test results, the ANOVA results did not suggest that the preference for unmasked faces diminished in the younger infant group.

Individual data (spaghetti plots in Figure 3) indicate that upright face preferences for masked and unmasked faces varied across age groups. For instance, younger infants' preferences tended to vary between masked and unmasked conditions, whereas preferences were relatively uniform between the conditions in older infants. As for older infants, many younger infants tended to prefer upright faces (scores above 0.5) in both the masked and unmasked conditions; however, several young infants tended to prefer inverted faces (scores below 0.5) in the unmasked condition. Based on these observations, we conducted additional analyses to examine the differences between age groups using nonparametric methods. To this end, we first categorized the variant subgroup behaviors into four categories: (1) showing a preference for both upright masked and unmasked faces; (2) showing a preference only for the upright masked face but not for the unmasked face; (3) showing a preference only for the upright unmasked face but not for the masked face; and (4) showing no preference for either upright face. We then divided the infants into the following four categories based on visual preference for each face condition: preference score greater than the chance level of 0.5 in both the masked and unmasked face conditions (++); preference score greater than 0.5 only in the masked face condition (+–); preference score greater than 0.5, only in the unmasked face condition (– +); and preference score smaller than 0.5 in both the masked and unmasked face conditions (––). Table 2 shows the number of infants in each preference category (++, + –, –+, or ––) × age group (4–5 or 7–8 months). A chi‐square test revealed no significant difference in the number of infants among all combination groups (*x* ^*2*^ [3] = 2.486, Cramer's *V* = 0.197). The results suggest there was no significant difference in the tendency of upright face preference between the younger and older infant groups. This was consistent with the ANOVA findings reported in the main analysis.

**TABLE 2:** The number of infants in each preference category (++, +–, –+, ––) and age group (4–5 months and 7–8 months).

	++	+–	–+	––
4–5 months	18	10	3	1
7–8 months	14	9	7	2

*Note:* The four preference categories were defined as follows: (++) infants who showed a preference score > 0.5 (random chance) in both the masked and unmasked face conditions, (+–) infants who showed a preference score > 0.5 only in the masked face condition, (+–), infants who showed a preference score > 0.5 only in the unmasked face condition, (––) and infants who showed a preference score < 0.5 in both the masked and unmasked face conditions.

## 4 Discussion

This study aimed to investigate the effect of wearing face masks on infant face perception during the COVID‐19 pandemic. We hypothesized that infants born during the pandemic who were frequently exposed to masked individuals would recognize masked faces as faces. Consequently, they would show a visual preference for the upright face in both the masked and unmasked face conditions. One‐sample *t*‐tests versus chance revealed a significant upright face preference in both the masked and unmasked face conditions among infants aged 7–8 months. This suggests that older infants perceived both masked and unmasked facial stimuli as faces. Interestingly, for infants aged 4–5 months, the same analysis revealed a significant upright face preference in the masked‐face condition but not in the unmasked condition. The finding of a significant upright face preference in the masked face condition in both age groups aligns with our hypothesis that infants born and raised during the COVID‐19 pandemic would process masked faces as faces; however, the lack of a significant upright face preference for unmasked faces in the younger group was unexpected.

Providing a plausible explanation for this unexpected finding in young infants is challenging. However, it is important to note that the results of both the main analysis (two‐way ANOVA) and additional analysis (chi‐square test) consistently indicated no significant difference in the overall tendency to show an upright face preference for masked versus unmasked faces between younger and older infants. Given that these analyses do not support the interpretation that younger infants exhibit a weaker upright face preference for unmasked faces than older infants, it is difficult to draw a firm conclusion from the non‐significant preference in the younger group. Moreover, while the 4–5‐month‐olds' preference for upright faces in the unmasked condition was not statistically significant (*p* = 0.056), the effect size (*g* = 0.64) suggests a meaningful trend. The magnitude of this effect was comparable to the significant preference found in their 7–8‐month‐old counterparts (*g* = 0.77), suggesting that the lack of significance in the younger group may be an issue of statistical power rather than the true absence of preference. Further investigations with larger sample sizes may be necessary to ascertain the robustness of this preference and its developmental trajectory.

Our second hypothesis was that the upright face preference for masked faces would be stronger for older infants, based on the assumption that they would have a greater experience of seeing people wearing face masks than younger infants. However, this hypothesis is not supported by the current results. In this study, the upright face preference was stronger in the masked condition than in the unmasked condition for both age groups. ANOVA revealed a significant main effect of the mask condition on the upright face preference scores, but there was no significant age × mask condition interaction effect. The stronger preference for upright masked faces compared to unmasked faces observed among infants in both age groups may reflect the effect of the COVID‐19 pandemic on the development of face perception. That is, owing to frequent exposure to masked faces during the pandemic, infants may have learned to recognize faces even when they were partially occluded by face masks. Whether a similar pattern of results occurs in infants not routinely exposed to masked faces remains an open question for future research.

The significant effect of age revealed by ANOVA indicated a weaker upright face preference in older infants than in younger infants, both in the masked and unmasked face conditions. Such an effect of age on the upright face preference in infancy has not been reported in previous lab‐based studies. For instance, Dobkins and Harms (2014) reported no significant difference in preference for upright faces between 4‐ and 8‐month‐old infants. Interestingly, a recent study using the same remote testing paradigm as the present study also found that the upright face preference was significantly weaker in older (9–11 months) than in younger (6–8 months) infants (Yamanaka et al. 2025). It is unclear why the upright face preference declined with age in these remote experiments. We suspect that remote experiments, in which the surrounding environment is less controlled, might interfere more with older infants compared with younger infants, resulting in noisier data for older infants. Further studies are required to clarify whether the age effect reflects methodological differences or true developmental trends.

As mentioned in the Introduction, several recent studies have examined the effect of face masks on face perception in infants during the COVID‐19 pandemic. Galusca et al. (2023) conducted an experiment similar to that involving masked‐face stimuli in the present study but obtained different results. They tested 3–12‐month‐old infants and reported that, overall, the infants did not show a significant preference for upright masked faces, and only older infants (aged 9–12 months) showed a significant preference for inverted masked faces. Unlike Galusca et al. (2023), we found a significant upright face preference in the masked‐face condition. One possible explanation for the discrepancy is the difference in the level of experience with masked faces among the infants enrolled. During the COVID‐19 pandemic, the Japanese government strongly recommended that facemasks be worn at all times in public spaces. During our data collection period (July 13, 2021, to November 7, 2022), and even thereafter, the mask‐wearing rate in Japan was very high (> 90%) (Kusama et al. 2023; Nagata et al. 2024). Thus, infant participants in this study had relatively few opportunities to see people without facemasks outside their homes. By contrast, they would have had many opportunities to adapt to masked faces, thus acquiring the ability to perceive masked faces as humans. However, given the lack of direct evidence of differences in experiences with masked faces between the participants in Galusca et al. (2023) and those in the present study, this possibility should be treated with caution. Other possible reasons for the conflicting results between these studies include differences in experimental design (onsite vs. remote), facial stimuli (photographs of white Caucasian females vs. computer‐generated Asian females/males), and various other factors (e.g., size, luminance, and color of the visual stimuli).

One limitation of this study is the lack of a direct comparison between the pre‐ and post‐pandemic periods. Therefore, definitive conclusions regarding the effects of the COVID‐19 pandemic and the associated use of face masks in public spaces on the development of face perception during infancy cannot be drawn. Conducting experiments with infants born after the COVID‐19 pandemic is important for future research to determine whether the significant upright masked face preference observed in this study was specific to infants raised during the pandemic.

## 5 Concluding Remarks and Future Directions

These results indicate that infants born during the COVID‐19 pandemic in Japan, who would have had many opportunities to acquire experience with masked faces, had an upright face preference, even for masked faces. Additionally, among all infants tested, the magnitude of preference was greater in the masked‐face condition than in the unmasked‐face condition, which may reflect the relative lack of opportunities for infants to see a variety of unmasked faces during the pandemic.

One question that remains unanswered is whether the stronger upright face preference for masked‐face stimuli weakens over time. Tracking the evolution of face perception in individuals born during the COVID‐19 pandemic is an important future task.

## Author Contributions

**Nobu Shirai:** conceptualization, methodology, software, validation, formal analysis, resources, writing – original draft, visualization, supervision, project administration, funding acquisition. **Mizuki Kawai:** conceptualization, methodology, validation, formal analysis, investigation, data curation, writing – review and editing, visualization. **Yumiko Otsuka:** conceptualization, methodology, writing – review and editing, funding acquisition. **Megumi Kobayashi:** investigation, resources, writing – review and editing, project administration, funding acquisition. **Tsunehiko Tanaka:** conceptualization, methodology, writing – review and editing, supervision.

## Funding

This study was financially supported by JSPS research Grants (23K22350 and 23H00076 to NS, 23K25752 and 24H00178 to MK, and 22K03212 to YO). We thank Mai Kasahara, Akiho Ono, and Nahoko Sato for their assistance with data collection.

## Conflicts of Interest

The authors declare no conflicts of interest.

## Data Availability

The data that support the findings of this study are available from the corresponding author upon reasonable request.
